# Changes in the Antioxidant Potential of *Camellia sinensis* Cultures under the Influence of Phenolic Precursors

**DOI:** 10.3390/molecules29020474

**Published:** 2024-01-18

**Authors:** Maria A. Aksenova, Tatiana L. Nechaeva, Evgenia A. Goncharuk, Maria Y. Zubova, Varvara V. Kazantseva, Petr V. Lapshin, Andrej Frolov, Natalia V. Zagoskina

**Affiliations:** K.A. Timiryazev Institute of Plant Physiology, Russian Academy of Sciences, 127276 Moscow, Russia; nechaevatatyana.07@yandex.ru (T.L.N.); mariia.zubova@yandex.ru (M.Y.Z.); k.v.-90@mail.ru (V.V.K.); p.lapshin@mail.ru (P.V.L.); frolov@ifr.moscow (A.F.); nzagoskina@mail.ru (N.V.Z.)

**Keywords:** tea plant, callus culture, L-phenylalanine, *trans*-cinnamic acid, naringenin, superoxide dismutase, peroxidase, flavanols, proanthocyanidins

## Abstract

The viability, productivity and survival of higher plants under the adverse factors influence are largely determined by the functional activity of the antioxidant system. The aim of our work was to investigate changes in formation of high-molecular (superoxide dismutase and peroxidase) and low-molecular (phenolics, including flavanols and proanthocyanidins) antioxidants in callus culture of *Camellia sinensis* under influence of phenolic precursors (L-phenylalanine—3 mM, *trans*-cinnamic acid—1 mM, naringenin—0.5 mM). According to the data obtained, the effect of precursors on tea callus cultures did not lead to significant increasing of superoxide dismutase and peroxidase activity in most cases. However, it led to the increased accumulation of the total phenolics content, as well as flavanols and proanthocyanidins contents. For *C. sinensis* callus cultures, the most promising regulator of phenolic compounds was L-phenylalanine, in the presence of which its content increased almost twice. Thus, the exogenous effect of various precursors is possible to use for the targeted regulation of certain phenolics classes accumulation in plant cells.

## 1. Introduction

Plants in the process of their growth and development are constantly exposed to various environmental factors, which leads to changes in their metabolism and functional activity [1]. This also applies to the antioxidant system, which protects them from the action of reactive oxygen species (ROS) such as superoxide radical (radical anion) O_2_^•−^, hydroperoxide radical HO_2_^•^, hydroxyl radical HO^•^, hydrogen peroxide H_2_O_2_, singlet oxygen ^1^O_2_ [2,3,4]. These molecules are formed under conditions of normal plant growth during respiration and photosynthesis; however, under stressful conditions, the balance between their formation and inactivation is disturbed. The accumulation of these molecules leads to the so-called “oxidative explosion”, causing changes in metabolism, initiation of pathological processes, necrotic damage to vegetative and generative organs and even death of plants [2,4].

Despite all these changes, plants remain viable, which is largely due to the functional activity of their antioxidant system [3]. Its main components are high-molecular and low-molecular antioxidants, which, being present in lower concentrations compared to the oxidized substrate, significantly delay or inhibit its oxidation [4].

High-molecular antioxidants of plant tissues are represented by various enzymes, which include superoxide dismutase (SOD), various peroxidases, catalase and other enzymes [3,4]. Among them, SOD scavenging the superoxide radical (O_2_**^•^**^−^) is considered to be the “primary” protector of cells from the action of ROS [5]. An important role is also assigned to peroxidases, the functional role of which is associated with the neutralization of hydrogen peroxide formed in cells—a very toxic compound for them [2,3].

Low-molecular antioxidants include various compounds of primary and secondary plant metabolism (carotenoids, tocopherols, polyamines, polyphenols, etc.) that can slow down or prevent the oxidation of other compounds [3]. Among low-molecular weight antioxidants, polyphenols or phenolic compounds (PCs)—compounds of secondary metabolism synthesized in all plant cells—have attracted great interest [6]. PCs, due to their chemical properties and interaction with ROS, are able to reduce their level, thereby affecting the redox mechanisms in plants and, as a consequence, maintaining their viability [7,8].

**Figure 1:**
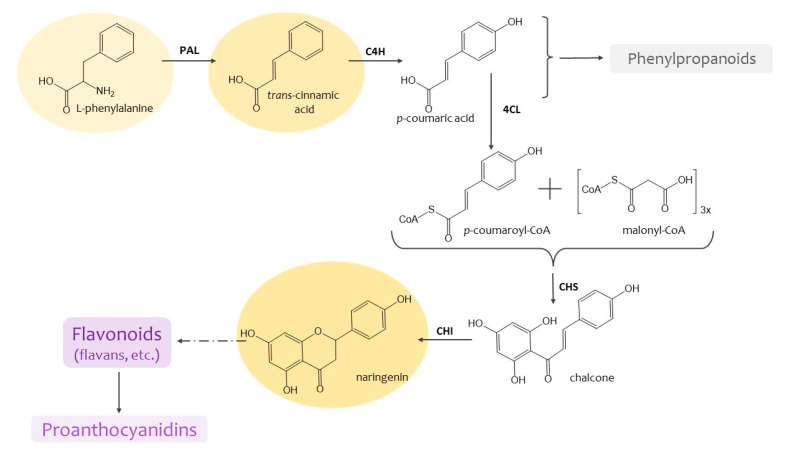
The main stages of phenolic compounds biosynthesis. PAL—L-phenylalanine ammonia-lyase; C4H—cinnamic acid 4-hydroxylase; 4CL—4-coumarate-CoA-ligase; CHS—chalcone synthase; CHI—chalcone isomerase.

Phenolics are extremely diverse in structure and chemical properties [9,10]. They are represented by different classes such as phenylpropanoids, flavonoids and proanthocyanidins [8]. Presently, great success has been achieved in the study of their metabolism: the sequence of their formation and the enzymes involved in these reactions have been established (Figure 1). The role of these metabolites in protecting against UV radiation, various pathogens, mechanical damage and resistance to various external influences is well known [6,9]. They are also involved in the formation of plant cell walls and may serve as storage [8]. The issue on the content and composition of the PCs metabolites is of great importance [3,8,9].

In general, the balance of high-molecular and low-molecular antioxidants ensures the viability of plants and their resistance to stress [2,4,6]. At the same time, if the level of high-molecular antioxidants is associated with the “protection” of cells from ROS, then the accumulation of low-molecular metabolites of phenolic nature is also of practical importance, since many of their representatives can be used as pharmacologically valuable drugs for the health of the population [11,12].

One of the promising approaches to the study of plant metabolism is plant cell and tissue cultures grown in vitro [13]. This is due to the fact that they represent a “simpler” biological system compared to a plant, which is grown under strictly controlled conditions and retains the specificity of the metabolism of an intact plant [14]. Using the action of various exogenous factors, it is possible not only to study the mechanisms of its regulation, but also to select conditions for increasing the productivity of cell cultures as potential producers of biologically active plant metabolites for pharmacology [13]. These include the action of biosynthesis precursors of various compounds, including PCs [13,15,16]. However, it is worth noting that the effectiveness of this action depends on the concentration of the compound, the duration of its exposure and the species-specific characteristics of cultures [17,18,19].

The tea plant (*Camellia sinensis* L.) is one of the unique plants with a specialized metabolism aimed at the formation of PCs [20,21,22]. It is known that the proportion of these secondary metabolites in its tissues can reach 30% by dry weight [23]. *C. sinensis* callus cultures also retain the ability to form PCs, although at a lower level (no more than 10% by dry weight) [24]. It should also be emphasized that the main components of their phenolic complex are flavans, or flavanols—substances with P-vitamin capillary-strengthening activity [25]. All this indicates that in vitro culture of the tea plant is not only a good model object for studying the regulation of phenolic metabolism, but also a potential producer of biologically active compounds.

Considering all of the above, the purpose of our work was to study the effect of the PCs precursors (L-phenylalanine, *trans*-cinnamic acid, naringenin) on the antioxidant potential of the *C. sinensis* callus culture, investigating changes in formation of high-molecular (superoxide dismutase and peroxidase) and low-molecular (phenolics, including flavanols and proanthocyanidins) antioxidants.

## 2. Results

### 2.1. Morphological Characteristics of Tea Cells Cultures

**Figure 2:**
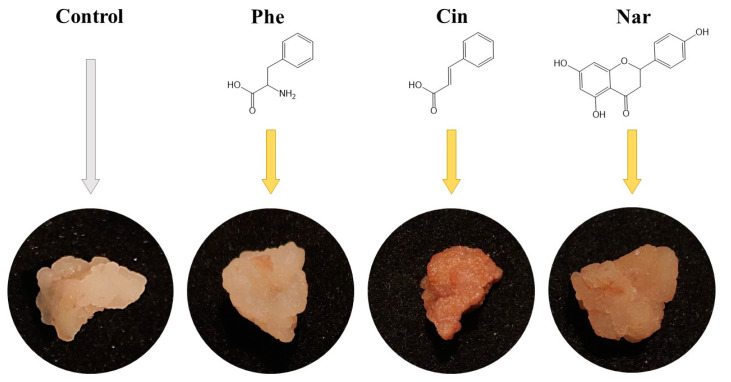
The tea cells cultures grown for 7 days on the liquid Heller culture medium of the basic composition (Control) or enriched with L-phenylalanine (Phe; 3 mM), *trans*-cinnamic acid (Cin; 1 mM) or naringenin (Nar; 0.5 mM).

Comparison of morphological parameters of in vitro plant cultures exposed to exogenous factors is an important indicator of assessing their physiological state [26]. The tea callus cultures grown in vitro in a liquid nutrient medium were compact, quite dense and had a beige color (Figure 2).

On the main nutrient medium or on the medium with additional L-phenylalanine (Phe), they had a light beige color. On the medium with naringenin (Nar), the calluses were darker in color, and on the medium with *trans*-cinnamic acid (Cin), they were denser and dark beige. All this indicates minor differences in the morphology of tea callus cultures grown on media with various precursors of PCs, relative to the control.

The water content determination in tea callus culture almost did not reveal significant differences between the variants (Table 1). One can only note a statistically significant increase in the water content of the culture under the Nar action.

### 2.2. The Antioxidant System of Tea Cells Cultures

#### 2.2.1. The High-Molecular Antioxidants in Tea Cells Cultures

**Figure 3:**
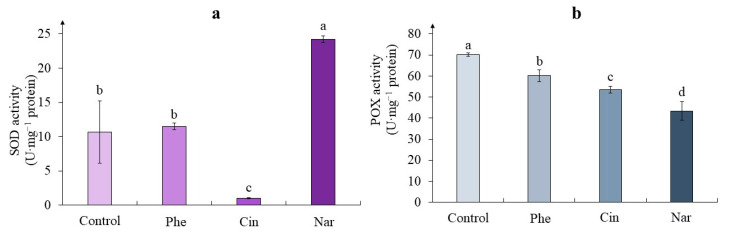
The activity of level of superoxide dismutase—SOD (**a**), and peroxidase—POX (**b**), in tea cells cultures grown for 7 days on the liquid Heller culture medium of the basic composition (Control) or enriched with L-phenylalanine (Phe; 3 mM), *trans*-cinnamic acid (Cin; 1 mM) or naringenin (Nar; 0.5 mM). Results are expressed as means ± SDs, *n* = 3. The significant differences at *p* < 0.05 are denoted by different Latin letters.

Determination of the superoxide dismutase (SOD) activity in tea callus cultures revealed statistically significant differences between the control and two experimental (Cin and Nar) variants (Figure 3a). The highest SOD activity was noted in calli grown on the medium with Nar, where it was almost 2.5 times higher than the control variant. The Cin effect was manifested at a very low level of the SOD activity in tea cells culture. As for the calli exposed to Phe, the enzyme activity was statistically equal to the control values. 

Our research also indicated that the peroxidase (POX) activity was the highest in tea cells culture of the control variant (Figure 3b). In the experimental variants, there was a statistically significant trend towards a decrease in POX activity in the series: Control-Phe-Cin-Nar. This decrease in tea cells culture grown on the medium with Phe amounted to 15%, on the medium with Cin, to 24%, and on the medium with Nar, to 40%.

#### 2.2.2. The Low-Molecular Antioxidants in Tea Cells Cultures

**Figure 4:**
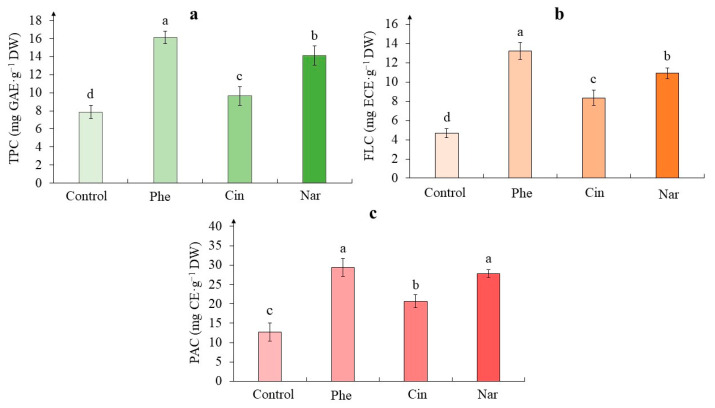
The total phenolic content—TPC (**a**), the flavanols content—FLC (**b**), the proanthocyanidins content—PAC (**c**), in tea cells cultures grown for 7 days on the liquid Heller culture medium of the basic composition (Control) or enriched with L-phenylalanine (Phe; 3 mM), *trans*-cinnamic acid (Cin; 1 mM) or naringenin (Nar; 0.5 mM). Results are expressed as means ± SDs, *n* = 3. The significant differences at *p* < 0.05 are denoted by different Latin letters.

According to the obtained data, when growing tea cells cultures on media with phenolic precursors, the total phenolic content was significantly higher than in the control (Figure 4a). The greatest increase in this parameter was noted on the medium with Phe when it exceeded the control data twice. In the presence of Nar, the content of PCs increased by 79%, and in tea cells culture treated with Cin—by 23%, relative to the control values.

As for the flavanols content, its lowest accumulation was noted in the tea cells culture of the control (Figure 4b), while, in all experimental variants, it was significantly higher. Thus, in the presence of Phe, its content exceeded the control value by 150%. A fairly close effect was observed with the action of Nar (an excess by 130%). In the Cin variant, this increase was 180%.

Determination of the proanthocyanidins content in tea cells cultures also showed the stimulating effect of all precursors (Figure 4c). Its highest amount was observed in calluses grown on the media with Phe and Nar, which almost equally exceeded the values of the control variant (2.3 and 2.2 times, respectively). At the same time, the action of Cin increased the content of these compounds only 1.6 times relative to the control variant.

### 2.3. The Phenylalanine Ammonia-Lyase Activity in Tea Cells Cultures

**Figure 5:**
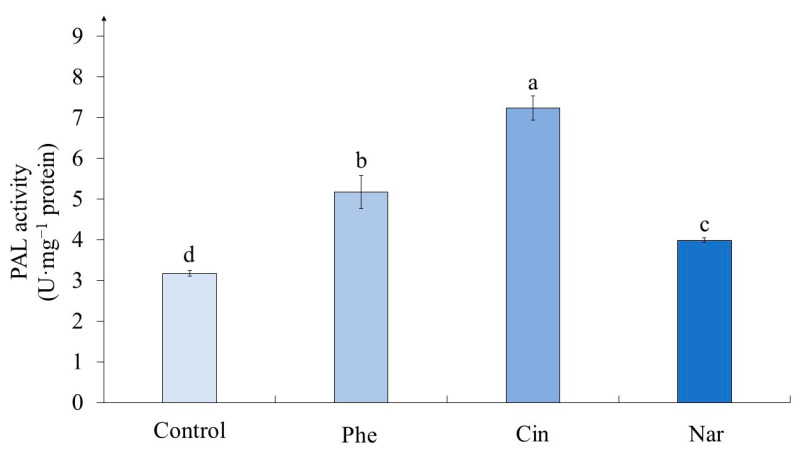
The level of phenylalanine ammonia-lyase (PAL) activity in tea callus cultures grown for 7 days on the liquid Heller culture medium of the basic composition (Control) or enriched with L-phenylalanine (Phe; 3 mM), *trans*-cinnamic acid (Cin; 1 mM) or naringenin (Nar; 0.5 mM). Results are expressed as means ± SDs, *n* = 3. The significant differences at *p* < 0.05 are denoted by different Latin letters.

In experimental variants, the activity of phenylalanine ammonia-lyase (PAL) exceeded that of the control and this effect depended on the precursor added to the nutrient medium (Figure 5). The greatest activity of the enzyme was noted in the callus on the medium with Cin, which exceeded the control value by more than two times (228%). On the medium with Phe, the enzyme activity in tea callus culture exceeded the control value by 63%, and on the medium with Nar—by 25%.

### 2.4. The Level of Lipid Peroxidation in Tea Cells Cultures

**Figure 6:**
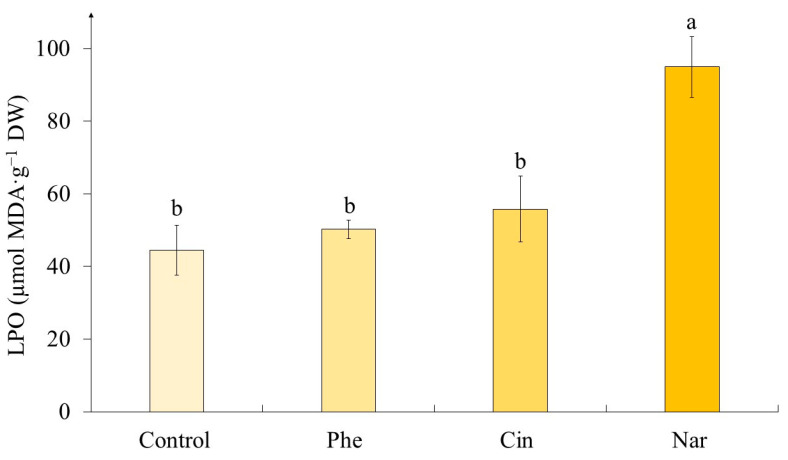
The level of lipid peroxidation (LPO) in tea cells cultures grown for 7 days on the liquid Heller culture medium of the basic composition (Control) or enriched with L-phenylalanine (Phe; 3 mM), *trans*-cinnamic acid (Cin; 1 mM) or naringenin (Nar; 0.5 mM). Results are expressed as means ± SDs, *n* = 3. The significant differences at *p* < 0.05 are denoted by different Latin letters.

According to our data, the lipid peroxidation (LPO) level was statistically equal in calluses of the control variant and those exposed to Phe and Cin treatment (Figure 6). The only exception was the variant with the action of Nar, where the level of LPO was twice as high as the control.

## 3. Discussion

### 3.1. Morphological Characteristics of Tea Cells Cultures

The water content is an important parameter of the viability of plant cells and tissues. It is worth noting that statistical processing of the data did not reveal a significant effect of treatment with different precursors on this parameter (Table 2).

However, there was a statistically significant increase (2%) in the water content of the culture under the Nar action (Table 1). It should be emphasized here that a change in this parameter within 5% does not indicate significant changes in the physiological processes of cells [15]. The described effect of Nar is confirmed by the data obtained in the work of Sharma et al. where a similar trend was noted [27]. At the same time, there was shown no change in water content using the example of *Carthamus tinctorius* seedlings in the presence of the indicated above precursor [28]. It is worth noting that for Phe, a similarity to our data effect was noted for the *Vitex agnus castus* shoot cultures and *Cicer arietinum* seedlings [29,30]. While the Cin effect on the example of *Pisum sativum* was the opposite and revealed in a decrease in water content [31]. 

Based on all of the above, it can be concluded that short-term exposure to various precursors of a phenolic nature did not cause significant changes in the morphological characteristics of tea callus cultures. Perhaps this effect is due to the regulation of their pro-/antioxidant balance, which is important in maintaining the viability of plant cells [32]. And in this case, an important role is assigned to the functioning of the antioxidant system in plant cells, the main components of which are high-molecular and low-molecular antioxidants [33].

### 3.2. The Antioxidant System of Tea Cells Cultures

It is known that an increased accumulation of ROS causes the development of oxidative stress due to an imbalance between the ROS generation and removal [3]. This leads to a number of pathological processes and plant diseases, necrotic damage to vegetative and generative organs, and even plant death [4]. The main damaging effects of ROS at the cellular level are damage to nucleic acids through the oxidation of deoxyribose, damage to organelles, peptide bond breaks, initiation of lipid peroxidation processes that cause an increase in membrane viscosity and disruption of diffusion processes and accumulation of damaged and self-aggregating proteins. At high levels of ROS accumulation, apoptosis or programmed cell death can occur [3,34].

In order to utilize ROS, plants are able to synthesize various types of antioxidants, enzymatic and non-enzymatic [34]. In this case, the functioning of the organism’s protective systems is of great importance, one of which is the antioxidant system, which includes numerous antioxidants that can slow down or prevent the oxidation of organic substances [3,35]. The antioxidant system functioning can be regulated by exposure to various exogenous compounds, including phenolic precursors, which is discussed in detail in the sub-chapters below [16,36,37].

#### 3.2.1. The High-Molecular Antioxidants in Tea Callus Cultures

Superoxide dismutase (SOD) is considered to be one of the main high-molecular antioxidants of plant cells and even the “primary” line of their protection against oxidative damage [3]. This enzyme prevents the oxidation of macromolecules by superoxide radicals [5].

According to the statistical processing, the different phenolic precursors treatment influenced the SOD activity (Table 3). Under the Cin effect the SOD activity did not exceed the control and even was minimal, which to a certain extent correlates with data on *Cucumis sativus* seedlings, where the absence of changes in the enzyme activity was noted [38]. As for the effects of Nar, there are several works confirming our data: a small but statistically significant increase in the activity of this enzyme was shown in *Carthamus tinctorius* seedlings, while this trend was more pronounced in *Phaseolus vulgaris* seedlings (almost a two-fold increase relative to the control) [28,37]. However, the Phe treatment did not result in any statistically significant changes in relation to control values.

High-molecular weight antioxidants also include peroxidases (POX) involved in the detoxification one of the ROS (H_2_O_2_) in plant cells [31,39]. According to the obtained data, the POX activity depended on the used precursor (Table 3).

Thus, our data indicate a decrease in POX activity when growing tea callus cultures on a medium with phenolic precursors. A similar trend was noted for *Phaseolus vulgaris* seedlings, in which, after the action of Nar, it decreased by almost three times relative to the control [37]. Using *Cucurbita ficifolia* seedlings as an example, it was shown that Cin treatment did not lead to statistically significant changes in POX activity, as well as Phe treatment, which did not affect this indicator in tomato leaves [38,40]. There are other data as well. Thus, the activity of this enzyme was significantly increased in *Fagopyrum esculentum* sprouts and in *Triticum aestivum* leaves under the action of Phe, as well as in tomato, *Pisum sativum* and *Cucumis sativus* seedlings under the action of Cin [16,31,38,39,41].

The tendency of each precursor individually on both enzymes’ activities should also be emphasized. The Phe treatment did not affect either, and the Cin action led to the decrease in both cases, while the Nar effect revealed in the low POX activity and in the highest SOD activity.

All of the above indicates that the Phe and Cin effects did not lead to an increase in the activity of SOD, whereas the presence of Nar in the culture medium led to a significant increase in the activity of this enzyme of the antioxidant system. Thus, we can say that the presence of phenolic precursors in the culture medium led to the changes in the activity of such high-molecular antioxidants as SOD and POX. It is worth noting that in the case of the latter enzyme, only a significant decrease in its activity was shown in all experimental variants. As for the activity of such a primary enzyme of the antioxidant system as SOD, the increase in its activity was characteristic only of the Nar treatment.

#### 3.2.2. The Low-Molecular Antioxidants in Tea Cells Cultures

PCs are one of the effective low-molecular antioxidants, which are considered the “second” line of defense of plant cells from the action of ROS [42,43]. Due to their chemical properties, they interact with active oxygen forms, thereby reducing their toxic effect on cells [9,10]. The statistical processing has shown that the content of different phenolics depended on the treatment with different precursors (Table 4).

Determination of the total phenolic content in tea callus cultures is an important indicator in assessing their ability to accumulate these secondary metabolites [44,45]. On the example of *Cicer arietinum* leaves and *Sequoia sempervirens* callus culture, the increase in its content was shown in the presence of Phe, which was similar to our data [35]. While in the cells of the *Moringa oleifera* plant this trend was significantly less pronounced, herewith in quinoa sprouts there was no statistically significant difference between the experimental and control variants [30,46]. There are data confirming the increase in the accumulation of PCs in *Carthamus tinctorius* and *Vigna radiata* seedlings in the presence of Nar, while in the first case this indicator increased almost two-fold [27,28]. 

The main components of the phenolic complex, not only of the tea plant, but also of the cultures initiated from it in vitro, are flavanols, which in recent years have also been considered substances with antioxidant activity [47,48]. According to the data described in the Section 2, the intake of all phenolic precursors into tea calluses contributed to the activation of the phenolic metabolism, which was accompanied by the accumulation of flavanols characteristic of them. At the same time, the most effective “regulators” of this process were Phe (the initial stages of biogenesis) and Nar—an important intermediate of the flavonoid pathway. The example of *Triticum aestivum* leaves also showed a significant increase in the content of flavanols in the presence of Phe [16].

The tea plant and the callus cultures initiated from it are also characterized by the formation of oligomeric forms of PCs—proanthocyanidins [48,49]. The data obtained by us on the action of PhA are confirmed in the work of Feduraev et al. on the example of wheat leaves [16].

As for the individual effect of each precursor, the upregulating effect of Phe was the most pronounced on all phenolic classes. Nar had a similar to Phe effect, which was a little bit less noticeable. While Cin treatment had the least expressed effect on these parameters.

All of the above indicates that the cultivation of tea callus culture on a medium with various precursors was accompanied by an increase in the accumulation of low-molecular phenolic antioxidants in them, including flavanols, substances with P-vitamin capillary-strengthening activity. The intensity of this process may depend on the activity of the enzymes involved in their biosynthesis.

### 3.3. The Phenylalanine Ammonia-Lyase Activity in Tea Cells Cultures

It is known that phenylalanine ammonia-lyase (PAL) is an important enzyme in the initial stages of the PCs biosynthesis [50]. Based on the above presented data about the effect of precursors on the accumulation of phenolic compounds in callus tea cultures, we analyzed its activity in all studied variants (Figure 5). The statistical processing of the data revealed a significant effect of treatment with different precursors on this parameter (Table 5).

In experimental variants, the activity of PAL exceeded that of the control and this effect depended on the precursor added to the nutrient medium. The greatest activity of the enzyme was noted in the callus on the medium with Cin, which exceeded the control value by more than two times (228%). A different trend was observed in *Cichorium intybus* calluses when PAL activity decreased significantly relative to control in the presence of Cin [51]. On the medium with Phe, the enzyme activity in tea callus culture exceeded the control value by 63%, and on the medium with Nar—by 25%. A similar effect of Phe on the PAL activity was observed in the leaves of *Ocimum basilicum*, *Triticum aestivum* and in the shoots of *Chenopodium quinoa* [16,52,53]. It is worth noting that it was significantly more pronounced relative to the control variant, whereas in the case of buckwheat sprouts, this increase was statistically unreliable [41]. And on the example of *Glycine max* seedlings, a statistically significant decrease in PAL activity in the presence of Nar was recorded [50].

It is worth noting that the highest enzyme activity was shown under the Cin treatment, although the phenolic compounds content was the lowest one under this precursor effect. Perhaps in this case, further pathways of biosynthesis of polyphenolic compounds, for example lignin and its derivatives, have been activated. This issue is a subject for further research.

Thus, the cultivation of tea callus culture on a medium with phenolic precursors is accompanied by the activation of the key polyphenol biosynthesis enzyme and, as a result, the accumulation of phenolic compounds in them.

### 3.4. The Level of Lipid Peroxidation in Tea Cells Cultures

Changes in the growing conditions of plant cells, including the administration of additional components into the nutrient medium, can cause changes in the functioning of their antioxidant system [37]. The criterion of this process is the level of lipid peroxidation (LPO), often assessed by the content of malondialdehyde (MDA) [28]. The statistical processing has shown that LPO level depends on the precursor treatment (Table 6). It is worth noting that there was a statistically significant change (increasing) in MDA content only under Nar action (Figure 6); in other cases, its content was equal to the control values.

The data obtained by us are confirmed in a variety of works. Thus, using lentil sprouts and tomato plants as an example, no statistically significant changes were shown in the LPO level under PhA exposure [40,54]. Also, Cin treatment did not lead to significant changes in this parameter in *Cucurbita ficifolia* seedlings and *Tanacetum parthenium* leaves [38,55]. However, a different trend was shown on the example of *Cucumis sativus* seedlings: in this case, the MDA content significantly exceeded the control values [38]. As for the action of Nar, which caused the increase in the LPO level, according to our data, different reactions of plant objects to the presence of this precursor are shown in other works. Thus, its effect did not lead to changes in this parameter in the seedlings of *Vigna radiata* and *Phaseolus vulgaris* [27,37]. Nevertheless, there are data on the Nar treatment of *Carthamus tinctorius* seedlings, which are similar to the results we obtained [28].

Thus, it can be concluded that the effect of precursors on the LPO level depends both on the nature of this molecule and on the species specificity of the object under study. Moreover, in the case of tea plant cells, the stress effect was shown only under the influence of Nar, which was not reflected in morphological parameters; however, a slight increase in water content was noted.

## 4. Materials and Methods

### 4.1. Plant Material and Experimental Conditions

A heterotrophic callus culture was the object of the study. It was obtained from the young shoots stem of a tea plant (*Camellia sinensis* L., Georgian variety). For its cultivation, Heller culture medium was used in the following composition: 5 mg/L 2,4-dichlorophenoxyacetic acid, 25 g/L glucose and 7 g/L agar was used for its cultivation [48]. The calluses were grown under the conditions of the growth cabinet at the IPP RAS (25 °C, relative air humidity 70%, darkness 24 h). The subcultivation duration was 39 days.

**Figure 7:**
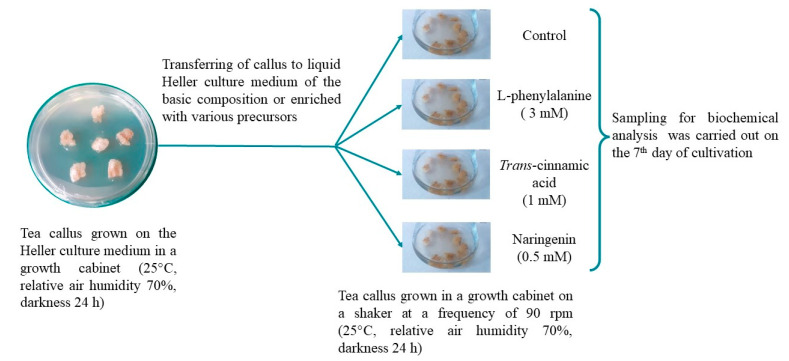
Experimental design.

Tea callus tissues (the weight of each callus was 220–250 mg) were placed in conical retorts (100 mL) to set up the experiment (Figure 7). Each retort contained the liquid Heller culture medium (15 mL) of the basic composition (control) or enriched with different phenolic precursors (Phe, 3 mM; Cin, 1 mM; Nar, 0.5 mM). The substances used in the experiment (Serva, Heidelberg, Germany) were dissolved in distilled water in advance, after that it was sterilized using membrane filters (MILLEX GV, 0.22 µm, Merck, Darmstadt, Germany) and added to the liquid Heller culture medium autoclaved beforehand. Concentrations of the precursors were chosen in preliminary experiments. The tea callus cultures were grown under conditions in the growth cabinet on a shaker at a frequency of 90 rpm (25 °C, relative air humidity 70%, darkness 24 h) and analyzed on the 7th day. The tea callus tissues were frozen with liquid nitrogen and stored at –70 °C for further biochemical analysis.

### 4.2. Determination of Morphological Characteristics of Tea Cells Cultures

To evaluate the morphological characteristics of tea callus cultures, such indicators as appearance, color and texture were used.

### 4.3. Determination of Water Content and Dry Weight in Tea Cells Cultures

The tea callus (150 mg FW each) was dried in the thermostat BD-115 (Binder, Tuttlingen, Germany) to a constant weight (70 °C, 48 h). Calculation of water amount and dry weight was carried out using the standard methods [56,57].

### 4.4. Determination of the Superoxide Dismutase Activity in Tea Cells

The superoxide dismutase (SOD) activity was evaluated by the level of inhibition by superoxide dismutase of the nitroblue tetrazolium (NBT) reduction reaction to formazane by superoxide radicals generating the riboflavin oxidation system [58]. To determine the enzyme activity, the frozen material (200 mg FW each) was homogenized in 0.067 M K, Na-phosphate buffer (pH 7.8) at a temperature of +4 °C. The homogenate was centrifuged at 7000× *g* for 20 min (centrifuge Minispin, Göttingen, Germany); the supernatant was used to determine the enzyme. The reaction was carried out by adding to 60 µL supernatant (enzymatic extract) 0.95 mL 0.1 M methionine, 0.32 mL 2 mM NBT and 0.23 mL 0.012 mM riboflavin [59]. The mixture was exposed to fluorescent light for 30 min. The solution absorbance was measured at 560 nm. It was expressed in U/mg protein. One SOD unit was defined as the amount of enzyme required to inhibit 50% of the nitro blue tetrazolium photoreduction in comparison with tubes lacking the plant extract [58,59]. The protein content was determined by the Bradford method [60].

### 4.5. Determination of the Peroxidases Activity in Tea Cells Culture

To determine the activity of peroxidases (POX), samples of frozen in liquid nitrogen tea callus culture (500 mg FW each) were homogenized in a 0.067 M K,Na-phosphate buffer (pH 7.8) with the addition of insoluble polyvinylpyrrolidone (50–75% by weight of fresh tissue; Serva, Heidelberg, Germany). The homogenate was centrifuged at 12,000× *g* for 20 min (centrifuge Minispin, Göttingen, Germany). The obtained supernatant was used to determine the enzyme activity. These manipulations were carried out at a temperature of +4 °C. The activity of POX was determined by the change in optical density (wavelength 470 nm) in a reaction mixture of the following composition: 650 µL of 5 mM hydrogen peroxide (Chimmed, Moscow, Russia), 650 µL of 4 mM guaiacol (Merck, Darmstadt, Germany) solution and 400 µL of the initial supernatant [61]. Reaction time was 3 min. The POX activity was estimated due to the formation of the reaction product, tetraguaiacol. The enzyme activity was expressed in U/mg protein [62]. The protein content was determined by the Bradford method [60].

### 4.6. Extraction of Phenolic Compounds from Tea Cells Culture

The extraction of the tea callus culture samples (50 mg FW each) frozen in liquid nitrogen was carried out using 96% ethanol [63]. The obtained homogenate was subjected to thermal treatment (45 °C, 30 min; thermostat Gnom, Moscow, Russia); then it was centrifuged at 12,000× *g* for 5 min (centrifuge Minispin, Göttingen, Germany). The precipitation was separated and the obtained supernatant was used to evaluate the content of different phenolic compounds applying spectrophotometric methods.

### 4.7. Determination of Different Phenolic Compounds Classes in Tea Cells Culture

The determination of the total phenolic content quantity was carried out with the Folin–Ciocalteu reagent (Panreac, E.U.) at 725 nm according to our modified method, using a four-point calibration curve of the gallic acid standard (Serva, Germany) [64]. The amount of flavanols was determined with a 1% vanillin (Merck, Germany) in 70% sulfuric acid at 500 nm, using a four-point calibration curve of the epicatechin standard (Serva, Germany) [24]. Proanthocyanidins were determined with butanol reagent (n-butanol:HCl, 95:5 *v*/*v*) at 550 nm, using a four-point calibration curve of the cyanidine standard (Sigma, Burlington, VT, USA) [65].

The total phenolic content was expressed in mg of gallic acid equivalents per g of dry weight (mg GAE·g^−1^ DW), the content of flavanols in mg of epicatechin equivalents per g of dry weight (mg ECE·g^−1^ DW) and the content of proanthocyanidins in mg of cyanidine equivalents per g of dry weight (mg CE·g^−1^ DW).

### 4.8. Determination of the L-Phenylalanine Ammonia-Lyase Activity in Tea Cells Culture

The L-phenylalanine ammonia-lyase (PAL) activity was determined by homogenization of the frozen material (200 mg FW each) in a 0.1 M Na-borate buffer (pH 8.8) containing 0.5 mM EDTA (Reanal, Budapest, Hungary) and 3 mM dithiothreitol (Reanal, Hungary) with the addition of insoluble polyvinylpyrrolidone (50% by weight of fresh tissue; Serva, Germany) [63]. The homogenate was filtered and centrifuged at 13,000× *g* for 40 min (centrifuge Minispin, Göttingen, Germany) and then the supernatant was used to determine the enzyme activity. These manipulations were carried out at a temperature of +4 °C. The PAL activity was determined by the change in optical density (wavelength 290 nm) in a reaction mixture of the following composition: supernatant, 0.1 M Na-borate buffer, 0.02 M L-phenylalanine in ratio 1:1:1 (Serva, Germany). The PAL activity was evaluated using the formation of trans-cinnamic acid from L-phenylalanine for 1 h. The PAL activity was expressed in U/mg protein [63]. The protein was analyzed according to Bradford [60]. 

### 4.9. Determination of the Level of Lipid Peroxidation in Tea Cells Culture

The level of lipid peroxidation was assessed by a reaction with thiobarbituric acid (Dia-m, Moscow, Russia) and content of its product—malonyl dialdehyde (MDA). Frozen plant material (200 mg FW each) was homogenized in 5 mL of 0.1 M Tris-HCl buffer (pH 7.5; Reanal, Hungary) with 0.35 M NaCl. Then the reaction of the obtained homogenate with 0.5% solution of TBA in a 20% aqueous solution of trichloroacetic acid was carried out. The reaction mixture was kept on a water bath for 30 min; the optical density of the solution was estimated at 532 nm [66]. A molar extinction coefficient equal to 1.56 × 10^−5^ M^–1^ cm^–1^ was used to count the MDA content (µmol MDA g^−1^ DW) [67].

### 4.10. Statistical Analysis

All studies were conducted in three biological and three analytical replications. Analysis of variance (ANOVA) was carried out with SigmaPlot 12.2 software (Technology Networks, Sudbury, UK). A Normality Test (Shapiro–Wilk) and all Pairwise Multiple Comparison Procedures (Holm-Sidak method) were used to perform mean separation. Different Latin letters indicate the significant differences (*p* < 0.05).

## 5. Conclusions

The antioxidant system plays an important role in preserving the vital activity of plants, as well as their adaptation to changing environmental conditions. Their main components are high-molecular and low-molecular antioxidants, the balance of which determines the presence of active forms of oxygen in the cell and their detoxification. At the same time, high-molecular antioxidants, represented by various enzymes, are considered the first line of defense, and low-molecular are considered the second and possibly, additional ones, the functional activity of which manifests itself at a later stage of exposure to various factors. And this thesis fully corresponds to the data we have obtained for tea cells cultures. After different precursors exposure there was a change in enzyme (high-molecular) antioxidants activity. Herewith, the SOD activity varied in two experimental variants: Cin treatment contributed to its lowest values while Nar treatment enhanced it. In the case of the POX activity, it was always lower under the precursors action than in the control variant. However, the accumulation of low-molecular antioxidants of phenolic nature increased, as well as the activity of the key enzyme of their biosynthesis—PAL. Perhaps it was the accumulation of these metabolites that served as the basis for maintaining the level of LPO in the most studied samples at the control level.

It is also important to note the fact that using various precursors of phenolic compounds as components of nutrient media for growing plant cell cultures, it is possible, to a certain extent, to regulate the accumulation of substances with antioxidant activity in them, in particular polyphenols. For tea callus cultures, the most promising regulator of their accumulation was Phe, in the presence of which their content increased almost two times and manifested itself not only at the level of the total content of these metabolites, but also at the level of flavanols—substances with P-vitamin capillary-strengthening activity.

Based on this, we consider it important and promising to study the regulation of phenolic metabolites under the action of precursors using a high-performance liquid chromatography method, as well as molecular genetic approaches. Of great interest is a more detailed study of the balance of high and low-molecular weight antioxidants.

## Figures and Tables

**Table 1:** Water content in tea cells cultures grown for 7 days on Heller’s liquid culture medium of the basic composition (Control) or enriched with L-phenylalanine (Phe, 3 mM), *trans*-cinnamic acid (Cin, 1 mM) or naringenin (Nar, 0.1 mM).

Variants	Water Content, %
Control	92.13 ± 0.02 ^b^
Phe	92.05 ± 0.25 ^b^
Cin	92.42 ± 0.94 ^b^
Nar	94.01 ± 0.23 ^a^

Results are expressed as means ± standard deviations, *n* = 3. The significant differences at *p* < 0.05 are indicated by different Latin letters.

**Table 2:** One-way ANOVA showing the effect of the precursor’s treatments on water content in *Camellia sinensis* callus cultures.

Sum of Squares	df	Mean Square	F	*p*
7.822	3	2.607	3.492	0.129

**Table 3:** One-way ANOVA showing the effect of the precursor’s treatments on the activity of superoxide dismutase (SOD) and guaiacol-dependent peroxidases (POX) in *Camellia sinensis* cells cultures.

Determination	Sum of Squares	df	Mean Square	F	*p*
*SOD activity*	42,575.27	3	14,191.76	17.212	0.005
*POX activity*	65.28	3	21.76	16.485	<0.001

**Table 4:** One-way ANOVA showing the effect of the precursor’s treatments on total phenolic compounds, flavanols, proanthocyanidins content in *Camellia sinensis* callus cultures.

Determination	Sum of Squares	df	Mean Square	F	*p*
*Total phenolic content*	245.598	3	81.866	17.096	<0.001
*Flavanols content*	132.252	3	44.084	8.811	0.001
*Proanthocyanidins content*	791.121	3	263.707	15.22	<0.001

**Table 5:** One-way ANOVA showing the effect of the precursor’s treatments on the activity of L-phenylalanine ammonia-lyase in *Camellia sinensis* callus cultures.

Sum of Squares	df	Mean Square	F	*p*
2218.885	3	739.628	47.576	<0.001

**Table 6:** One-way ANOVA showing the effect of the precursor’s treatments on the lipid peroxidation level in *Camellia sinensis* callus cultures.

Sum of Squares	df	Mean Square	F	*p*
4702.636	3	1567.545	10.176	0.004

## Data Availability

The data presented in this study are available on request from the corresponding author.
